# Clinical educators’ attitudes towards the use of technology in the clinical teaching environment. A mixed methods study

**DOI:** 10.1002/jmrs.335

**Published:** 2019-04-21

**Authors:** John McInerney, Ruth Druva

**Affiliations:** ^1^ Department of Medical Imaging and Radiation Sciences Faculty of Medicine, Nursing and Health Sciences School of Primary Health and Allied Health Care Monash University Melbourne Victoria Australia; ^2^ Radiology Department Royal Melbourne Hospital Parkville Melbourne Australia

**Keywords:** Attitude, education, educational technology, medical, radiography

## Abstract

**Introduction:**

In healthcare, there is ongoing flux in expectations for students and practitioners. Establishing integrated systems of monitoring and evidencing students’ development is imperative. With current trends towards the use of technology in tertiary education, online learning environments (OLEs) could constitute more effective evidencing of student progress in the clinical environment. However, there is little research exploring clinical educators' experiences with implementing technology in clinical education.

The research aimed to:

- Examine clinical educators’ attitudes towards technology and its use in clinical education.
- Explore clinical educators’ experiences of implementing technologies in a clinical environment.

**Methods:**

A mixed methods approach was taken to explore the aims. A previously validated technology attitude survey (TAS) was used with slight modifications, as well as open‐ended qualitative responses. These explored clinical educators’ experiences of the implementation of one specific OLE (PebblePad™) in their clinical environments. The survey was sent to clinical educators involved in the supervision of Medical Imaging students on clinical placement.

**Results:**

Clinical educators play pivotal roles in students’ professional development and, given current trends in tertiary education, are under increasing pressure to utilise OLEs. This poses particular challenges in clinical environments. Irrespective of the challenges, successful implementation of technology in any environment is dependent on the attitudes of the users.

**Conclusions:**

Clinical environments have specific challenges when implementing technology such as access to computers and time constraints on practitioners. Even with positive attitudes towards technology, a change in pedagogical outlook when using technology in clinical teaching is necessary.

## Introduction

In healthcare, there is ongoing flux in expectations for students and practitioners. There has been an explosion of technology and the volume of medical knowledge has increased exponentially.^1^, ^2^, ^3^

In Australia, the Medical Radiation Practice Board of Australia (MRPBA) professional capabilities framework^4^ obliges radiographers to embrace contemporary conceptualisations of competence. They must keep abreast of shifting expectations^5^, ^6^ while the profession struggles to shed the perception that it is fulfilling the role of technical operatives.^1^ Due to these shifting expectations and to assure the MRPBA that students meet registration requirements on graduation; it is imperative to establish integrated systems of monitoring and evidencing students’ development. With trends towards the use of technology in tertiary education^7^, ^8^, ^9^ online learning environments (OLEs) could fulfil this need.

Within radiography, while course content is increasingly facilitated online^10^, ^11^, recording radiography students’ development during clinical rotations remains traditional.^10^ However with technological trends, clinical educators are increasingly pressured to utilise OLEs.^12^, ^13^ Introducing technology in clinical environments poses challenges for clinical educators as much as it does for students and teachers.^12^, ^14^ These range from; cost, security of the devices and the data on them and pedagogical challenges.^13^ Negative attitudes towards technology is a barrier to successful implementation of technology in clinical education.^14^, ^15^, ^16^, ^17^

While the attitudes of academic staff, clinical staff in non‐teaching roles and students to technology has been explored^18^, ^19^, ^20^, ^21^ much less is known about clinical educators’ perceptions of technology.^13^, ^22^

The Bachelor of Radiography and Medical Imaging (Honours) at Monash University is a 4‐year integrated academic and clinical course. Intake is approximately 80 students yearly. Students complete clinical placements each year. The degree provides a qualification allowing students to seek employment in Australia and worldwide. In 2014, PebblePad™ was introduced as a contemporary learning platform for clinical studies, replacing paper workbooks.

PebblePad™ is a web‐based platform offering an array of tools to help students record evidence of their learning and reflect on their clinical experiences. PebblePad™ and its ePortfolio functionality provides students with a holistic and integrated learning experience with a focus on preparation for professional life. Students can continue to access the platform after graduation. The implementation of PebblePad™ requires significant input from clinical educators.

### Aims of the study

The research aimed to:

- Examine clinical educators’ attitudes towards technology generally.
- Examine clinical educators’ perceptions of the use of technology in clinical education.
- Explore clinical educators’ experiences of implementing PebblePad™ in their clinical environment.

For the purpose of this study, ‘technology’ refers to computers, software, hardware and use of the Internet, in keeping with Maag's study^17^ using a similar survey tool.

## Methods

This mixed methods study reports on clinical supervisors, educators and tutors responsible for the supervision of Medical Imaging students on clinical placement. Herein, they will be referred to as ‘clinical educators’. Quantitative data meant we could measure the prevalence of the dimensions of the phenomenon we were exploring. Qualitative questions allow deeper exploration of a phenomenon where little is known about it, which was the case in this instance.^23^

An email invitation was sent by the Clinical Support Officer to clinical educators to participate. It included an explanatory statement and a link to the survey. Sites with minimal student engagement, for example only taking students occasionally, were excluded. This was done to keep the data clean, only collecting responses from educators who are au‐fait with course expectations.

### Data collection

Data collection was conducted between May and July 2017 using an online survey through Qualtrics™. The decision to complete the anonymous questionnaire ultimately rested with respondents.

The first section of the survey collected demographic data.

The second section provided quantitative data. This was minimally modified from a validated Technology Attitude Survey (TAS). Mc Farlane et al^24^ tested the TAS for reliability and validity to appraise teachers’ attitudes towards technology. The TAS was modified by Maag^17^ for the student nursing setting and likewise found adequate reliability for the tool. Permission was granted from Maag to modify the TAS for this study.

The TAS was adapted to ensure it was fit for purpose. One statement, ‘Technology makes me feel stupid’, was removed as it was irrelevant. ‘Nursing student’ was replaced by ‘clinical educator’ to represent the audience.

The modified TAS contained 14 items with 5‐point Likert scale responses. Questions were based on positively and negatively geared statements portraying positive and negative attitudes towards technology. This ‘reverse wording’ changes the direction of the scale by asking the same or similar questions in a positive and negative voice and adds to the validity of responses, reducing response sets and bias.^25^

The third section of the survey, modelled by the researchers, evaluated clinical educators’ experiences with the use of PebblePad™ in the clinical setting. There were two quantitative and two qualitative questions. The quantitative questions evaluated clinical educators’ experiences with using PebblePad™ in the clinical setting. These appraised how easy the clinical educators’ found it to learn the platform as well as how difficult the implementation was for them. One of the qualitative questions evaluated for perceived challenges using OLEs in a clinical setting. The other explored what clinical educators consider the advantages of OLEs over paper‐based documentation.

### Data analysis

Quantitative data were initially explored using statistical descriptive analyses in SPSS™. For the purpose of data analysis the ordinal scale responses to positively geared question response were scored (5‐Strongly agree, 4‐Agree, 3‐Neither agree nor disagree, 2‐Disagree, 1‐Strongly disagree). Negatively geared statements were reverse scored.

An independent samples t‐test was used to determine whether location, rural or remote, was significant in influencing attitudes towards technology. All the quantitative data, the TAS and PebblePad™ questions, were included in this analysis.

A Pearson's’ correlation coefficient was computed on the TAS questions to assess the relationship between clinical educators’ attitudes towards technology generally and their perceptions of its use in their role as educators. A scatter plot summarises the results to illuminate the interplay between the two variables. To conduct this analysis the eight TAS questions relating to technology generally were considered a single variable vs. the six TAS questions relating to the perception of the use of technology in clinical education. The sum of mean scores were calculated, computed and plotted in SPSS™.

Thematic analysis, using Braun and Clarke's^26^ method, was used to interpret the qualitative data. It provides a rich and detailed interpretation and there is an emphasis on reflexive dialogue which the researcher engages in throughout the process before reporting the themes. It involves a six‐step approach (Table 1) and the researcher moves forward and back between the steps as many times as required to make sense of the data.^26^

**Table 1:** Braun and Clarke's six phases for thematic analysis.^26^

Phase 1: Familiarise with the data	Phase 4: Review themes
Phase 2: Generate codes	Phase 5: Define themes
Phase 3: Search for themes	Phase 6: Name and write up themes

### Ethics approval

Ethics approval was granted by the Monash University Human Research Ethics Committee, project number 0197.

## Results

From a pool of 74 possible participants, 49 surveys were returned. One survey was incomplete and excluded from the data analysis. This constitutes a response rate of 48/74, 65%. There was a 100% response rate within the surveys to both the qualitative and quantitative components.

The majority of respondents worked in Metropolitan sites (37/48, 77.1%). 39/48, 81.3% worked at a single clinical site. Medium sized sites were best represented 24/48, 50% with a relatively even mix of large 14/48, 29.2% and small 15/48, 31.3% sites represented. A majority of respondents worked in private imaging 29/48, 60.4% (Table 2).

**Table 2:** Participant demographic characteristics

Characteristic	*n*/*N*	%
My clinical teaching site is		
Rural	11/48	23
Metropolitan	37/48	77
My clinical teaching site/s is^a^		
Large	14/48	29
Medium	24/48	50
Small	15/48	31
My clinical teaching site is		
A public institution	19/48	40
A private institution	29/48	60
I work at		
One clinical teaching site	39/48	81
Two clinical teaching sites	6/48	13
>Two clinical teaching sites	3/48	6

a Multiple responses possible.

**Figure 1:**
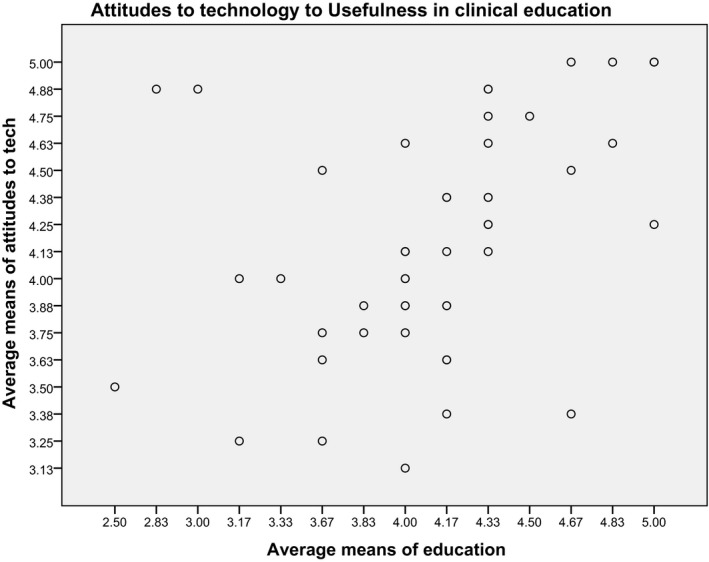
Correlation of attitudes towards technology versus the perceived usefulness in clinical education.

Using the modified TAS, participants were asked eight questions based on their attitudes towards technology generally (Table 3). These appraise personal feelings when using technology such as confidence, nervousness and perceived importance and level of difficulty in learning new technologies. Six questions were based on their perceptions of the use of technology in clinical education (Table 4). A general positive attitude towards technology was evident. A Pearson's r showed a mildly positive correlation between attitudes towards technology and an appreciation for the benefit of technology to the role of clinical educator (Table 5). A scatter plot illustrates that the relationship between them is not absolute (Fig. 1).

**Table 3:** Descriptive analysis, attitudes to technology

	Strongly disagree (%)	Disagree (%)	Neither agree nor disagree (%)	Agree (%)	Strongly agree (%)	Mean
Q 6. I like using technology	0	2	6	52	40	4.29
Q 7. I feel confident with my ability to learn technology	2	0	0	46	52	4.46
Q 8. Learning about technology is worthwhile	0	0	0	42	58	4.58
Q 9. Working with technology makes me feel nervous	23	46	25	6	0	3.85
Q 12. I'm not the type of person to do well with technology	33	54	13	0	0	4.21
Q 14. I find that I need to work hard to learn about technology to master it	17	50	21	10	2	3.69
Q 15. Using technology is difficult for me	35	44	19	2	0	4.13
Q 17. I feel uncomfortable using most technology	35	50	10	2	2	4.15

**Table 4:** Descriptive analysis, perceived use of technology in clinical education

	Strongly disagree (%)	Disagree (%)	Neither agree nor disagree (%)	Agree (%)	Strongly agree (%)	Mean
Q 5. Knowing how to use technology is a necessary skill for me as an educator	0	0	6	38	56	4.5
Q 10. I use my knowledge of technology in many ways as a clinical educator	2	0	21	58	19	3.92
Q 11. Technology is important to my role as clinical educator	0	6	13	52	29	4.04
Q 13. I can appreciate how technology can be used to facilitate clinical learning	0	0	4	71	25	4.21
Q 16. Knowing about technology can make me a better educator	0	6	15	58	21	3.94
Q 18. Technology really won't assist me in my role as an educator	21	56	17	2	4	3.88

**Table 5:** Pearson's correlation: attitudes to technology and its perceived use in clinical education

Characteristic	Mean	Pearson's *r*
Attitudes to technology	4.17	0.363
Perceived use of technology in clinical education	4.08	

Two questions related specifically to the PebblePad™ platform (Q19 and 20). These were aimed at appraising how easy the clinical educators found it to learn how to use the platform and how difficult the implementation of it in a clinical environment proved. There was a large spread in these responses (Table 6). 33.2% found PebblePad™ easy to learn how to use with 47.9% finding that it was not easy. Furthermore implementing the OLE in the clinical site was challenging for 35.4% of respondents, with only 37.5% finding it easy. This correlates with what Nairn et al^12^ cite as challenges facing clinical educators surrounding OLEs.

**Table 6:** Learning how to use PebblePad™ and implementation of PebblePad™ in a clinical environment

	Response on 5‐point Likert scale				
	Strongly disagree (%)	Disagree (%)	Neither agree nor disagree (%)	Agree (%)	Strongly agree (%)
Q 19. I found it quite easy to learn how to use PebblePad™	6	42	19	21	13
Q 20. I found the implementation of PebblePad™ in the clinical site relatively easy	15	21	27	33	4

No significant difference was noted between rural or remote locations (Table 7).

**Table 7:** TAS and rural and metropolitan location

Characteristic	*n*/*N* (%)	Average mean (SD)	*P* value
Metropolitan	37/48 (77)	4.05 (0.45)	0.661
Rural	11/48 (23)	3.98 (0.46)	

In keeping with Braun and Clarke's^26^ thematic analysis, the qualitative data were coded with five themes identified.

### Theme 1: availability of IT resources (hardware)

The strongest theme was lack of access and availability of IT resources for using OLEs. Respondent 5 quoted the ‘lack of availability of portable devices with multiple users’ at their site. This was compounded by the fact that ‘Access to computer time can be limited in a clinical practice that utilises computers for clinical work’ Respondent 27.

### Theme 2: time resource in clinical environment

A large proportion of participants reported that lack of time is a major factor when using OLEs in clinical sites. Respondent 24 mentioned that the ‘time to concentrate on filling out reports without interruption’ is difficult to find. Respondent 8 commented that ‘It is still easier to have a piece of paper in front of your when you are in the process of assessing a student’. Other time factors specific to OLEs was the time required to retrieve passwords and the time it takes to login.

### Theme 3: platform design

Respondents reported both favourably and unfavourably under this theme. Respondent 12 mentioned that ‘Difficult to navigate systems make documentation difficult’. Five respondents commented that the design of PebblePad™ was not intuitive to use. Respondent 6 suggested that it was ‘not designed for the purpose and so is very unintuitive to use’. Conversely four respondents mentioned the ease of navigating the platform over paper. Respondents 21 and 8 found it much easier ‘Not having to flick through pages of student books to find the correct page’.

This equivocal response was reflected in the quantitative data where 47.9% of respondents agreed with the statement ‘I found PebblePad™ easy to learn how to use’, with a further 18.7% saying they neither agreed nor disagreed with the statement.

Several respondents mentioned the capability for multiple users at different locations to be able to access the student's work as a significant advantage of the online platform. Respondent 11 mentioned that it was ‘Easy for all parties to access the online documents at the same time or at any time that it is convenient’. Respondent 41 furthered this, mentioning that ‘It is easier for me to check on the work at my own leisure without needing to chase anyone up or be told they left their book at home’.

### Theme 4: tracking of progress/documentation

There was a diversity of responses under this theme. This major theme has been broken down into two ‘sub’ themes for clarity. 13/48, 27.1% of respondents mentioned one or other of these sub themes

1. Tracking of the documentation and;
2. Tracking of student progress.

#### Tracking documentation

Participants mentioned that with an OLE one ‘can track documentation’ (Respondent 12) with ‘No misfiling or misplacement of paper documents’ (Respondent 39). Respondent 7 mentioned that having students’ work stored online allows ‘Easy storage and retrievals’ of documentation. Others mentioned that important documentation such as ‘Assessment and course structure guidelines for tutors are more available online’ (Respondent 33).

#### Tracking of student progress

24/48, 50% of respondents suggested that ‘there is a record of when and where assessments were performed and also no loss of information’ (Respondent 23). This allowed for ‘Real time checking of student progress’ (Respondent 26), stakeholders have ‘immediate access to the student's progress and my reports and can see quickly if the student is entering all the clinical requirements at the appropriate times’ (Respondent 25).

There was also a strong sense for longitudinal development. The online system made it ‘Easy to review and compare to previous work where appropriate’ (Respondent 40). ‘Sharing information across sites/placements allows for more adequate follow‐up on student's progress’ (Respondent 46) allowing for ‘more permanent record of progress in time relevance – make progress tracking easier’ (Respondent 47). Respondent 27 went on to record that the online version is a ‘Permanent record’ which could actually ‘be maintained and continually updated throughout [a radiographer's] career’

### Theme 5: increased security

Clinical educators were cognisant that OLEs are a more secure environment which is ‘Tamper proof’ (Respondent 41). Respondent 42 said that ‘my signature can't be forged … their work can't be tampered with and more importantly that they aren't able to take advantage of staff members who can't be bothered to check their work and sign everything off for them’.

## Discussion

Due to current trends in tertiary education, clinical educators are under pressure to utilise OLEs, posing challenges for clinical mentors.^8^, ^12^ This aspect of OLEs, that is in clinical environments, therefore requires attention. This study provides insight into clinical educators’ attitudes towards technology, both in general and specifically in clinical education. It explores some of the challenges faced by clinical educators when implementing such technology in the clinical environment.

In the current era, it can be taken for granted that computers are almost ubiquitously available. The research highlighted that this is not the case in clinical environments. The extent to which it seems computer allocations are so sparse was surprising, it should be explored whether there are differences between metropolitan and rural sites in this regard. While it was acknowledged that access to computers might be an obstacle, during the PebblePad™ roll out (2014–2015) clinical educators were offered tablet computers to address this challenge. Only 12 clinical sites took up the offer at the time. Thus further strategies should be considered to allow clinical educators with educational roles access to computer resources. This could involve protected time on computers, allocation of computers for the purpose etc. This would need to be considered in close collaboration with the clinical partners and their institutions.

Busy practitioners find themselves with time pressures and competing priorities when fulfilling teaching duties. There was mixed opinion whether the online environment aided efficient time management. For some, working online was quicker but for others, the necessity for passwords added to the time required making it more arduous than using paper versions. Disparity was also noted in how easy the educators found it to learn using PebblePad™ itself. There were some findings that could explain these differences. A ‘Lack of basic knowledge or skills’ (Respondent 41) and ‘My lack of IT skills as a mature radiographer’ (Respondent 33) were factors in adapting to OLEs. As Respondent 18 reported ‘Online can be more efficient when all involved have a certain level of competence’. This reinforces Chow, Herold, Choo & Chan's^27^ findings that training and support specific to technologies used in teaching is crucial for successful implementation. While some respondents mentioned that face to face training would be preferential, ‘I think that if you have not had any immediate instruction on how to use it, it can be a little difficult’ (Respondent 18), the geographical spread of clinical sites is challenging in this regard. Not all educators can attend hands on workshops on campus and the University staff required for in‐services is infeasible. It was reassuring that a higher proportion of respondents found they could implement PebblePad™ reasonably easily at their clinical sites (Table 6). This suggests that, despite the difficulties learning a new platform, educators were able to mitigate these during implementation.

The tracking of students’ progress within placements and over time was an important correlation with the vision for transitioning online. With paper workbooks, providing formative feedback on work in progress was difficult and it was also difficult to appreciate longitudinal development of skills and self‐efficacy in the students’ learning journey. The results from the study indicate that the objectives and vision for moving online has had some traction. As respondent 42 mentions, ‘Being able to actually view their work allows me to give them additional feedback in regards to their thoughts and what they write down’. There was an appreciation for the ability to track students’ progress over time rather than within single placements. This can help identify at risk students and assist educators to devise individual learning plans for those students while allowing appropriate follow‐up on student's progress.

Clinical educators found the online system more secure than paper workbooks. This is in keeping with what has been our own experience. Supervisors were confident that it was not possible for the students to tamper with the entries in the online environment. This is a positive of having password protection which can however add to the time pressure when using OLEs.

Respondents displayed a positive attitude towards technology in general. However responses to statements addressing the perceptions of using technology for clinical learning suggest that the link between knowing about technology and using it for a specific purpose is not absolute. While 100% of respondents said that learning about technology is worthwhile and 91.7% ‘like using technology’, there was a much more lukewarm response as to whether they saw the value of using technology in their roles as clinical educators. This begs the question of what clinical partners perceive as the role of technology in clinical teaching and how to successfully use it in supporting students on clinical placement. As Klenowski^28^ pointed out, clinical teachers need to change their teaching styles to balance traditional didactic approaches with contemporary conceptualisations of learning using technology. Klenowski^28^ further states that if the pedagogical approach to technology for clinical teachers is not addressed, technical reductionist approaches that trivialise the process of learning can make the learning superficial.

A further corroboration was that some respondents mentioned that they ‘see the students taking notes and then doubling up time by entering the information online’ Respondent 8. However this is a trend which evolved with paper workbooks and is not in keeping with the pedagogy of reflective practice. While the workbooks are the students’ primary submission they are expected to only carry a notebook with them on clinical rotations and following reflection on action, decide which cases will form the basis of their clinical portfolio (See * below).

*‘*Take notes/collect information continuously throughout placements in **notebook** or similar. Any new information regarding particular examinations should be written down in this book as it is effectively a learning outcome. When completing examinations on this region you can then implement this learning outcome and include in case write‐up’*. *(Excerpt from Clinical Studies Manual for Students,* 29 *2016, pp 38)*

One other finding worthy of mention was a comment from respondent 26, ‘a student may be at computer undertaking clinical documentation but is perceived by staff as being disinterested in clinical work’. This suggests that staff perceptions about the use of technology is manifest. As trends towards using technology in tertiary education accelerate this is a key finding. It is important for those who drive change to be aware of the landscape and perceptions of those at the coal face. These were deemed significant findings as it may be that there is a need to enculturate acceptance of technology, as well as teaching students about the appropriate use of technology on clinical placements.

## Implications for future research

This pilot study establishes a baseline but more importantly paves the way for further research in this crucial area. Incongruence exists between attitudes to technology generally and perceptions of the usefulness of technology for clinical education. A larger study should be completed to fully examine the significance of this finding. Furthermore while the research question aimed to examine the attitudes and experiences of clinical educators, it would be of great benefit to gain an understanding of the factors that influence clinical educators’ attitudes and experiences with technology in their environments. Factors such as the setting where the educators work and the nature of their appointments would be important to consider however were beyond the scope of this study. Educators have varied supervisory arrangements and appointments in different locations and it is feasible that these factors could influence their experiences and understanding of OLEs.

## Conclusion

Monash University was the first undergraduate radiography course in Australia to implement an OLE for all aspects of clinical placements. Clinical education is crucial in the education of Radiography students and educators assume a key role in this. PebblePad™ is at the heart of the clinical programme, requiring significant input from clinical educators. This study serves to give voice to this important but under represented group, those clinical educators tasked with implementation of OLEs in clinical environments. It is imperative to understand the perceptions of clinical educators within their environments. As evidenced by the study, even with a positive attitude to technology, clinical environments have particular challenges. With technology taking a more central role in education, it is imperative that we understand how to ensure it is utilised to its full potential in all domains of education. Therefore enculturating positive attitudes towards technology and associated pedagogical change is important. Training and support specific to OLEs is crucial for successful implementation. Partaking in this study will afford clinical educators an opportunity to reflect on their own experiences with technology in their roles and implementation strategies for future technological advances.

## Conflict of Interest

The authors declare no conflict of interest.

## References

[1] Baird M . Towards the development of a reflective radiographer: Challenges and constraints. Biomed Imaging Interv J 2008; 4: E9. 21614320 10.2349/biij.4.1.e9 PMC3097707

[2] O'Malley PA . Profile of a professional. Nurs Manage 2008; 39: 24–48. 10.1097/01.NUMA.0000320635.66188.fd. 18545218

[3] Wolff A , Pesut B , Regan S . New graduate nurse practice readiness: Perspectives on the context shaping our understanding and expectations. Nurse Educ Today 2010; 30: 187–91. 19699561 10.1016/j.nedt.2009.07.011

[4] Medical Radiations Practice Board . Professional capabilities for medical radiation practice, 2013 Australia [cited 2017 August 10]. Available from: http://www.medicalradiationpracticeboard.gov.au/Registration/Professional-Capabilities.aspx.

[5] McInerney J , Baird M . Developing critical practitioners: A review of teaching methods in the Bachelor of Radiography and Medical Imaging. Radiography 2016; 22: e40–53. 10.1016/j.radi.2015.07.001.

[6] Mason R , Williams B . Using ePortfolio's to assess undergraduate paramedic students: A proof of concept evaluation. Int J High Educ 2016; 5: e146–54. 10.5430/ijhe.v5n3p146.

[7] John‐Matthews JS , Gibbs V , Messer S . Extending the role of technology enhanced learning within an undergraduate radiography programme. Radiography 2013; 19: 67–72.

[8] Mackay BJ , Anderson J , Harding T . Mobile technology in clinical teaching. Nurse Educ Pract 2017; 22: 72–6. 10.1016/j.nepr.2016.11.001 27871040

[9] Kregor G , Breslin M , Fountain W . Experience and beliefs of technology users at an Australian university: Keys to maximising e‐learning potential. Australas J Educ Technol 2012; 28: 1382–404.

[10] Kowalczyk NK . Perceived barriers to online education by radiologic science educators. Radiol Technol 2014; 85: 486. 24806051

[11] Wertz C , Hobbs D , Mickelsen W . Integrating technology into radiologic science education. Radiol Technol 2014; 86: 23–31. 25224084

[12] Nairn S , O'Brien E , Traynor V , Williams G , Chapple M , Johnson S . Student nurses’ knowledge, skills and attitudes towards the use of portfolios in a school of nursing. J Clin Nurs 2006; 15: 1509–20. 17118073 10.1111/j.1365-2702.2005.01432.x

[13] O'Connor S , Andrews T . Mobile technology and its use in clinical nursing education: A literature review. J Nurs Educ 2015; 54: 137–44. 25693246 10.3928/01484834-20150218-01

[14] Chow S , Chin W , Lee H , Leung H , Tang F . Nurses’ perceptions and attitudes towards computerisation in a private hospital. J Clin Nurs 2012; 21: 1685–96. 22081971 10.1111/j.1365-2702.2011.03905.x

[15] Johansson P , Petersson G , Nilsson G . Nursing students’ experience of using a personal digital assistant (PDA) in clinical practice‐An intervention study. Nurse Educ Today 2013; 33: 1246–51. 22999410 10.1016/j.nedt.2012.08.019

[16] Selim H . Critical success factors for e‐learning acceptance: Confirmatory factor models. Comput Educ 2007; 49: 396–413. 10.1016/j.compedu.2005.09.004.

[17] Maag M . Nursing students’ attitudes toward technology: A national study. Nurse Educ 2006; 31: 112. 16708034 10.1097/00006223-200605000-00007

[18] Kennedy G , Judd TS , Churchward A , Gray K , Krause K . First year students’ experiences with technology: Are they really digital natives? Australas J Educ Technol 2008; 24: 108–22.

[19] Keller C , Cernerud L . Students’ perceptions of e‐learning in university education. J Educ Media 2002; 27: 55–67.

[20] Mahdizadeh H , Biemans H , Mulder M . Determining factors of the use of e‐learning environments by university teachers. Comput Educ 2008; 51: 142–54.

[21] Le‐May‐Sheffield S , McSweeney JM , Panych A . Exploring future teachers’ awareness, competence, confidence, and attitudes regarding teaching online: Incorporating blended/online experience into the “teaching and learning in higher education” course for graduate students. Can J High Educ 2015; 45: 72–14.

[22] Regan K , Evmenova A , Baker P , et al. Experiences of instructors in online learning environments: Identifying and regulating emotions. Internet High Educ 2012; 15: 204–12. 10.1016/j.iheduc.2011.12.001.

[23] Creswell J , Plano‐Clark VL . Designing and Conducting Mixed Methods Research, 2nd edn. SAGE Publications, Los Angeles, 2011.

[24] McFarlane T , Green K , Hoffman E . E.R.I.C. teachers’ attitudes toward technology: psychometric evaluation of the technology attitude survey. US Dept. of Education, Office of Educational Research and Improvement, Educational Resources Information Center, 1997.

[25] Coleman C , Bandalos D , DeMars C , Finney S , Pastor D . Effects of negative keying and wording in attitude measures: a mixed‐methods study 2013; ProQuest Dissertations and Theses.

[26] Braun V , Clarke V . Using thematic analysis in psychology. Qual Res Psychol 2006; 3: 77–101. 10.1191/1478088706qp063oa.

[27] Chow M , Herold DK , Choo TM , Chan K . Extending the technology acceptance model to explore the intention to use second life for enhancing healthcare education. Comput Educ 2012; 59: 1136–44.

[28] Klenowski V . Developing Portfolios for Learning and Assessment: Processes and Principles. Routledge Falmer, London, 2002.

[29] Monash University . Clinical studies manual for students. Monash University Clinical Documents. 2016.

